# Inter-Sensor Calibration between HY-2B and AMSR2 Passive Microwave Data in Land Surface and First Result for Snow Water Equivalent Retrieval

**DOI:** 10.3390/s19225023

**Published:** 2019-11-18

**Authors:** Shuo Gao, Zhen Li, Quan Chen, Wu Zhou, Mingsen Lin, Xiaobin Yin

**Affiliations:** 1 Key Laboratory of Digital Earth Science, the Aerospace Information Research Institute, Chinese Academy of Sciences, Beijing 100094, China; gaoshuo@radi.ac.cn (S.G.); lizhen@radi.ac.cn (Z.L.); 2 College of Resources and Environment, University of Chinese Academy of Sciences, Beijing 100049, China; 3 National Satellite Ocean Application Service, Beijing 100028, China; zhouwu@mail.nsoas.org.cn (W.Z.); mslin@mail.nsoas.org.cn (M.L.); 4 Beijing PIESAT Information Technology Co., Ltd., Beijing 100195, China; yinxiaobin@piesat.cn

**Keywords:** HY-2B, passive microwave, calibration, AMSR2, snow water equivalent

## Abstract

The self-designed HaiYang-2B (HY-2B) satellite was launched on 24 October 2018 in China at 22:57 UT in a 99.34° inclination sun-synchronous orbit. The Scanning Microwave Radiometer (SMR) on the core observatory has the capability to provide near-real-time multi-channel brightness temperature (Tb) observations, which are designed mainly for improving the level of marine forecasting and monitoring, serving the development and utilization of marine resources. After internal calibration and ocean calibration, the first effort to retrieve land surface snow parameters was performed in this study, which obtained extremely low accuracy both in snow extent and snow mass. Accordingly, land inter-sensor calibration was carried out between SMR and the Advanced Microwave Scanning Radiometer 2 (AMSR2) in order to broaden the research and application of SMR data on the Earth’s land surface. Finally, we evaluated the consistency of the snow extent and snow mass derived from the initial and land-calibrated SMR data. The results indicated that a systematic SMR cold deviation whose magnitude depends on the channel is present for all the compared channels. After intercalibration, the conformity of the snow extent and snow mass were substantially improved compared to before; the relative bias of the snow extent and snow mass decreased from −49.97% to 2.97% and from −51.71% to 3.01%, respectively.

## 1. Introduction

Geophysical parameters including snow depth/snow water equivalent (SWE), soil moisture, sea ice concentration, and precipitable water vapor are key quantities in the study of terrestrial water cycles and play an important role in the study of Earth energy balance [1,2,3,4]. The Earth system is a complex, dynamically changing system. Researches on the changing trends of the Earth system rely on long-term, stable, high-quality datasets of surface parameters [5,6]. Long-term sequence observations of surface data are of great significance for studying changes and predictions in the Earth’s environment [7,8]. 

Spaceborne passive microwave sensors play an important role in Earth observation, providing valuable data for retrieving a various range of critical land-surface parameters and studying changes in the Earth’s environment for time series [9]. With the development of remote sensing technology, more and more Earth observation satellites have been launched into space. The HaiYang-2B (HY-2B) satellite built by China was launched into sun-synchronous near polar orbit on 24 October 2018 at 22:57 UT (25 October at 6:57 A.M. China Standard Time) at the Taiyuan Satellite Launch Center, opening a new journey for the construction of the first marine dynamic environment monitoring network of the world. The Scanning Microwave Radiometer (SMR), serving as a geophysical observatory of water cycles, on the HY-2B satellite provides a direct link to the radiometer sensor constellation [10,11,12] and has the capability to obtain near-real-time brightness temperature for monitoring water resources of the land surface, tracking the extreme weather changes and other social applications. 

The Advanced Microwave Scanning Radiometer for Earth Observing System (AMSR-E) was operated from June 2002 to October 2011 in orbit on NASA’s Aqua satellite, while the AMSR-E instrument ceased the prescribed operations because the spin function of the rotating antenna failed in October 2011 [13]. The Microwave Scanning Radiometer 2 (AMSR2) sensor onboard the Global Change Observation Mission–Water 1 (GCOM-W1) mission successfully replaces the AMSR-E sensor [11]. The AMSR2 brightness temperature data have a wide range of applications for land-surface products and geophysical retrievals, including surface air temperature, snow cover area, snow depth/snow water equivalent, soil moisture, sea ice concentration, and near-surface soil freeze–thaw status in depicting regional patterns, monitoring natural climate changes, and tracking the global water cycle [14,15,16,17,18,19,20]. These products have been enhanced through continuous assessment and improvement to support global water, surface energy budget, and carbon cycle research [21,22,23].

Researchers at the National Satellite Ocean Application Service (NSOAS) have completed internal calibration and ocean calibration for SMR radiometers, based on which we have done the land calibration, in order to broaden the research and application of data on the Earth’s land surface. The cross-sensor calibration as an effective pattern further strengthens the natural resource satellite system, which facilitates the use of consistent long-term cross-platform data records for enhancing the ability of distinguishing global environmental changes [24]. From a principle point of view, the same geophysical parameter retrieval rules can be applied from AMSR2 to SMR sensors because they have similar sensor configurations including frequency, bandwidth, and incidence angle. However, there is always a slight difference between the brightness temperature data obtained from different radiometers, since spaceborne passive microwave sensors have differing instrument design characteristics, calibration systems, observing times, and non-overlapping operational records. Even in spaceborne remote sensing instruments with consistent design characteristics, such as Moderate Resolution Imaging Spectroradiometer (MODIS) mounted on the Terra and Aqua, the consistency of the observation data still needs to be verified by cross-calibration [25]. Therefore, careful cross-comparison and cross-calibration of the SMR and AMSR2 data records must be given to ensure the reliability, accuracy, and consistency of observations for consistent geoscience parameter retrievals when long baselines data are required.

Cross-sensor intercalibration is critical for land calibration to ensure the continuity of observational data records and quantify the changes in the environment of the Earth. Cross-platform calibration and validation have been performed for a long time related to many similar radiometers, including the Scanning Multichannel Microwave Radiometer (SMMR) carried on the Nimbus-7 [26], the Special Sensor Microwave/Imager (SSM/I) and Special Sensor Microwave Imager/Sounder (SSMI/S) carried on the Defense Meteorological Satellite Program (DMSP) [27,28,29,30], the Microwave Radiation Imager (MWRI) carried on the FengYun (FY) series satellite [31,32], and the AMSR-E and AMSR2 in various regions [33]. Jezek et al. compared the SMMR and SSM/I passive microwave data, and the results indicated that a general anomaly shift between sensors was apparent [28]. Evaluation of SWE from the Meteorological Service of Canada (MSC) showed that estimates derived from the SMMR were systematically and significantly lower than retrievals from the SSM/I when no cross-platform brightness temperature adjustments were employed [26]. Dai et al. developed the inter-sensor calibration between the SSM/I and SSMI/S and found that cross-calibration improved the consistency of the snow depth and the estimated snow cover area products in China [27]. Cavalieri et al. performed intercalibration for SSM/I and SSMI/S in order to obtain long-term sea ice datasets and found slight differences among the SSMI series sensors and SSMI/S [30]. Although the deviation was small, differences in sea ice extent and total ice cover area between the two platforms were found to be statistically significant [34]. Chen et al. compared the brightness temperature data of MWRI carried on FY-3B and AMSR-E with similar observation times in the polar ice cover area [32]. Du et at. developed an empirical approach for the intercalibration of microwave brightness temperature records over land from AMSR-E and AMSR2 using overlapping observations from the MWRI [33].

In this study, we first explored the radiometric performance of the SMR onboard HY-2B based on the first two months of on-orbit operations in Section 2, including the instrument design and data sets used in this study, and described the methodology of the data processing in detail. In Section 3, we developed a cross-platform calibration between the brightness temperature data from the SMR and AMSR2, and the predicted values of the deviation changes with the SMR brightness temperature were discussed in detail. In Section 4, we systematically assessed the consistency of daily snow extent and snow water equivalent in mid-latitude and high-latitude regions on a time series.

## 2. Data and Methods

### 2.1. Data Sets

In this section, the SMR and AMSR2 passive microwave brightness temperature data and the high spatial resolution Moderate Resolution Imaging Spectroradiometer (MODIS) land sets data, which are used to correct for forest attenuation for snow water equivalent, are introduced. To avoid the effects of snow melting liquid water in the snowpack on the snow cover identification, just cold overpass brightness temperature data were compared and used to produce the snow water equivalent datasets.

#### 2.1.1. Passive Microwave Brightness Temperature

The SMR is a nine-channel, five-frequency passive microwave radiometric system onboard the HY-2B satellite. The SMR measures atmospheric, ocean, and terrestrial microwave brightness temperatures on a global scale at 6.925, 10.7, 18.7, 23.8, and 37 GHz. Except for the 23.8 GHz-only vertical polarization channel, the other four frequencies have both vertical and horizontal polarizations. The SMR uses a process of matching three sets of electromagnetic wave feed sources on a parabolic antenna, of which 6.925 GHz and 10.7 GHz, 18.7 GHz and 23.8 GHz respectively share a set of dual-frequency feed sources, and 37 GHz uses a single-frequency feed source. The channel footprint varies with the channel frequency position during the scanning along the scan or track direction (Table 1). The SMR L2A_TB swath archive dataset includes antenna temperatures recorded across a 1600 km conical scan, Earth surface position for per pixel, satellite ephemeris, and radiometric calibration. The SMR swath data were obtained from NSOAS. This paper analyzed the SMR brightness temperature in land surface and tried to generate the snow cover area and snow water equivalent based on the early 73 days of on-orbit measurements. 

The AMSR2 instrument that is onboard the GCOM-W1mission was launched by Japan Aerospace Exploration Agency (JAXA) on 18 May 2012 [11]. The frequency bands include 6.925, 7.3, 10.65, 18.7, 23.8, 36.5, and 89.0 GHz. The sensor provides near real-time passive microwave observations from a height of approximately 700 km above the Earth’s surface, for which the equatorial overpass times are 01:30 and 13:30, respectively. The official AMSR2 radiometric accuracy is ±1.5 K [35], and the results from intercalibration between AMSR2 and other similar microwave radiometers based on radiative transfer computations, demonstrate that Tb measured by AMSR2 exhibits no apparent seasonal variation, with an average difference of 2–3 K in most channels compared to the Tropical Rainfall Measuring Mission Microwave Imager (TMI) and AMSR-E and a maximum difference of approximately 5 K [36]. Considering the uncertainty in large-scale footprint and the diversity of land surface cover, it’s reasonable to believe that AMSR2 has good radiometric performance. So, in this study, we take the effort to correlate SMR with AMSR2, as the first step for SWE estimation using SMR measurements. The AMSR2 data used in this paper were gridded with 0.25 degrees by 0.25 degrees pixels. The AMSR2 data were downloaded from JAXA’s G-COM official website http://suzaku.eorc.jaxa.jp/. The radiometric characteristics of the SMR and AMSR2 are provided in Table 1.

#### 2.1.2. Forest Cover Data

Snow depth and snow water equivalent retrievals from passive microwave remote sensing data will be influenced by vegetation, especially the dense forests. For vegetation treatment, the snow depth retrieval Foster (1997) algorithm [37] reduces the impact of vegetation by introducing the forest fraction. The latest MODIS/Terra Land Cover Type Yearly L3 Global 500 m SIN Grid (Short Name: MOD12Q1) dataset in 2017 (https://modis.gsfc.nasa.gov/data/dataprod/mod12.php) was used for fractional forest cover. These data are projected to geographic coordinates. From the land-cover map, five types of forest cover including evergreen needle leaf, evergreen broadleaf, deciduous needle leaf, deciduous broadleaf, and mixed forest were derived. In this study, a fractional forest cover ancillary file was derived from the original International Geosphere-Biosphere Program (IGBP) classification, where each data point is the forest fraction of 0–100%. For each ~500 m pixel, forest fraction percentage was obtained. These data were gridded to the 0.25° grid domains based on the geographic coordinates. The forest cover fraction data within each pixel of the passive microwave remote sensing data were obtained according to these forest cover data. The forest cover fraction was used to reduce the forest influence for snow water equivalent retrieval from passive microwave brightness temperature data.

### 2.2. Methods

The concrete time period when both SMR and AMSR2 data were acquired extends from 30 October 2018 through 31 December 2018. As of now, the SMR data have not been fully opened to the public. The data for this period are the longest data that we can apply for after the SMR on-orbit operation, and data collection is steady in the research area in which there were no erroneous scans. Before the cross-calibration, the brightness temperature data need to be preprocessed. First, the SMR L2A_TB swath data were processed according to geocoded data from the three feed sources of the sensors based on the requirements of grid projection, for which the daily averaged data were binned and gridded into 0.25 degrees by 0.25 degrees pixels in order to be consistent with the projection of the AMSR2 brightness temperatures. Second, the spliced grid data were revised for radiation to become brightness temperature values by using the conversion parameters provided by the radiometer. The final product is a series of daily maps of brightness temperature of the global scope. Third, taking the impact of the land surface water cycle process into account, a mask that includes water body, snow, and ice part was produced by the global land-cover map calculated from MOD12Q1 data. After the mask was applied, the observations of the study areas were screened out in the data processing.

**Figure 1:**
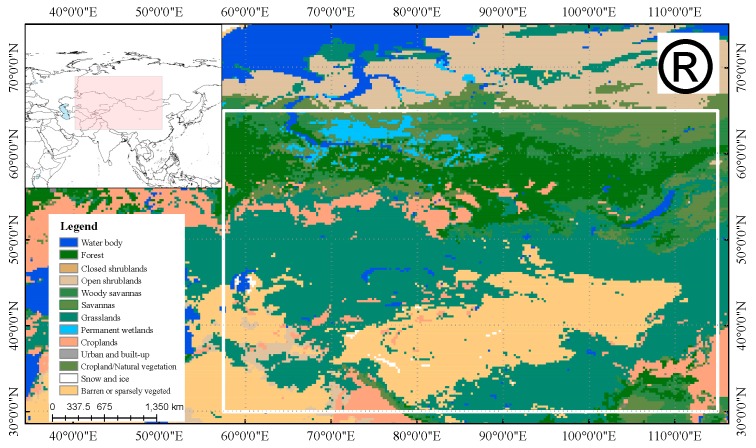
Land-cover map of the study area. The rectangular polygon is the study area that denotes transition from SMR to AMSR2 brightness temperature.

**Figure 2:**
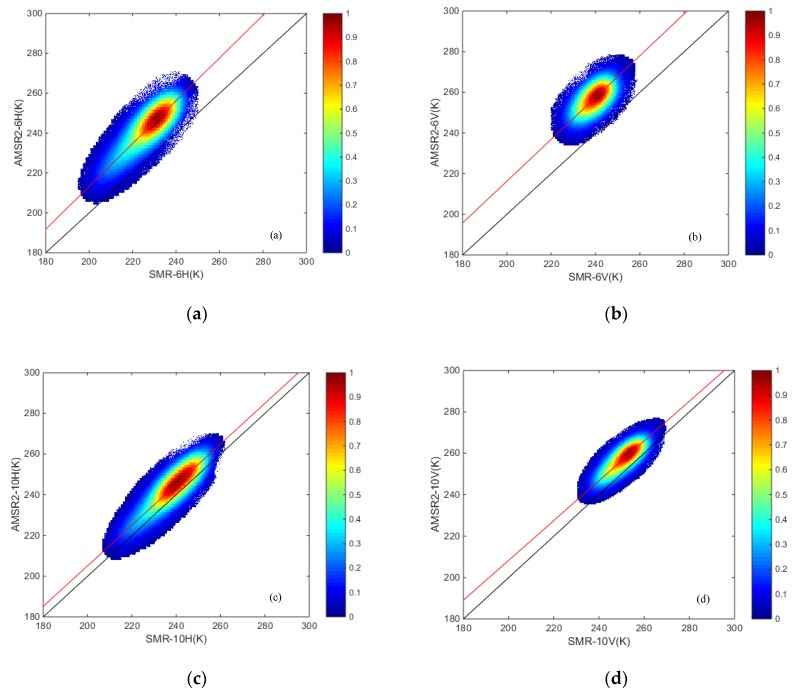

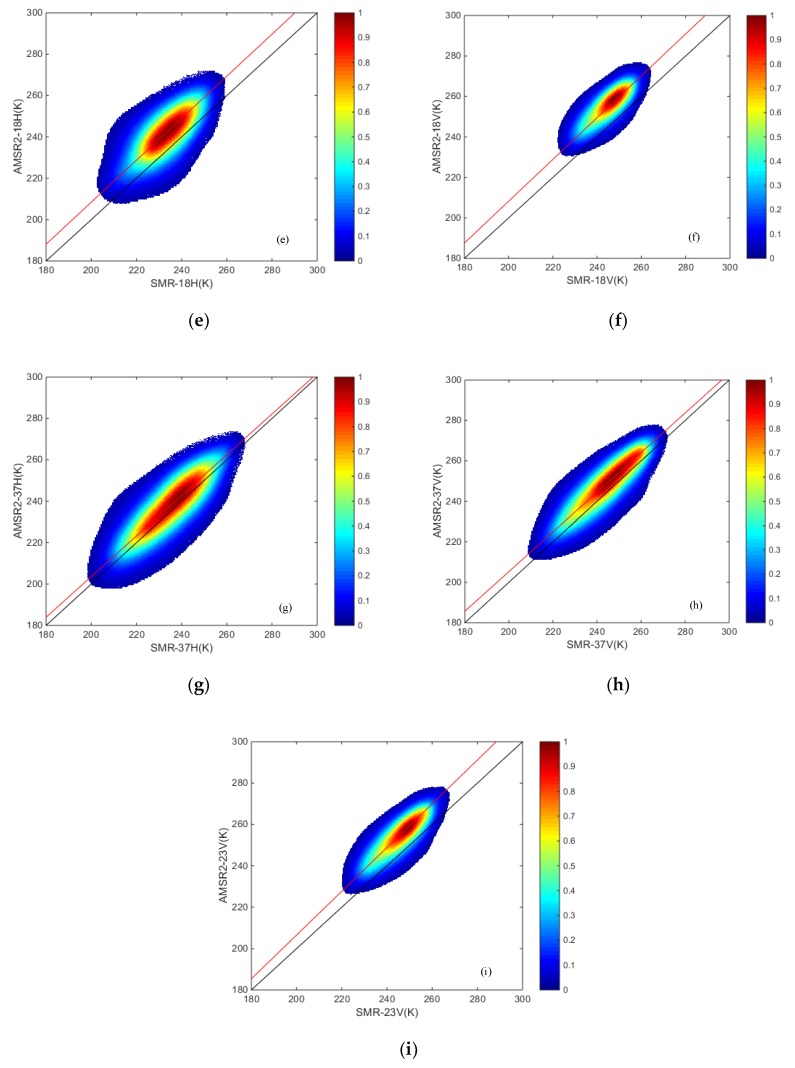
Density scatter plots between the brightness temperatures derived from the SMR and AMSR2: (**a**) 6H; (**b**) 6V; (**c**) 10H; (**d**) 10V; (**e**) 18H; (**f**) 18V; (**g**) 37H; (**h**) 37V; and (**i**) 23V.

As summarized in [24], the typical and effective approach involved in sensor intercalibration activities is to use linear regression to identify the relationship of brightness temperatures between sensors. In this paper, the regression method follows that of [26,27] and allows data cross-calibration in mid-latitude and polar regions. Based on existing research results for the cross-calibration of other sensors, the slopes of the correction equations were close to one, and the intercepts were close to zero. Thus, in our research, we a priori assume that the consistency between the brightness temperatures from SMR and AMSR2 is high. Scatterplots of the daily brightness temperatures at 6, 10, 18, and 37 GHz in both the horizontal and vertical polarizations and at 23 GHz in the vertical polarization from SMR and AMSR2 over the research area (Figure 1) during the overlapping period were plotted respectively (Figure 2). We produced a global land-cover map of 0.25 degrees by 0.25 degrees pixels based on MOD12Q1 data. The study area covers a wide range of continents, and the underlying surface features are rich in all types of IGBP classification, which makes sampling measurements have a high universal applicability to the inversion of terrestrial geophysical parameters (soil moisture, snow depth/snow water equivalent, etc.). In this paper, for increasing the universality of the research, we set a wide range of terrestrial research area, resulting in a large amount of observation data in each channel. The density threshold method [38], which is very efficient for the great amount data, was used to screen out the observation pairs for comparison. In order to obtain the accurate relationships between the brightness temperature data in the land surface, the density threshold method was used to avoid the impact of some abnormal points, including observations at the junction of water and land, and observations caused by other causes. The density threshold method removes the anomaly points by calculating the density of points within a certain radius around each point and setting a threshold. In this study, attempts were made to calculate the density in the range of 1 K radius; that is, the number of falling point pairs in the circular range whose surrounding radius is 1 K was calculated for each observation data point pair, and the threshold number was set to be 30. For each point pair, the number of points falling within a circular range of 1 K radius greater or equal to 30 was reserved for the following correlation analysis, and less than 30 were ignored. This design not only ensures the relationship trend of the overall data (the pairs of points selected by each frequency account for more than 98% of all data points), but also eliminates some points that could be observed abnormally.

In our research, approximately 27,000 common pixels were retrieved each day for each channel. As a result, there was a total of nearly 1,500,000 data pairs for each band for comparison, and linear regression coefficients were calculated. The common projection of the SMR and AMSR2 data results in a spatially precise comparison. However, the offsets in overpass time between sensors create a temporally imprecise scenario, since temperature and moisture effects at the land surface may influence the magnitude of brightness temperatures [26].

## 3. Results

### 3.1. Pairwise Comparison between SMR and AMSR2

**Figure 3:**
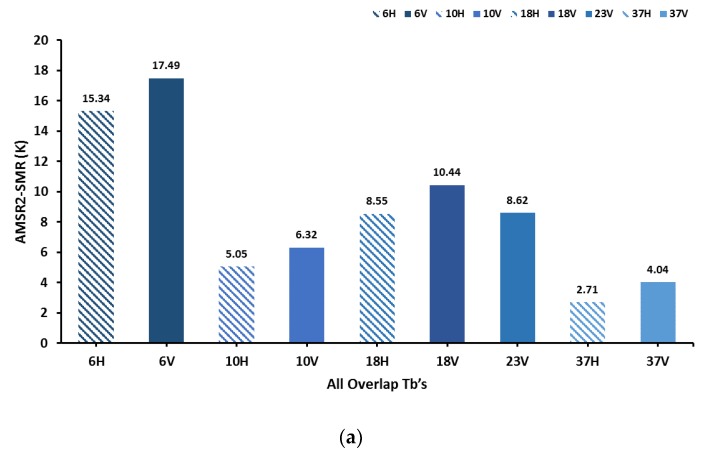

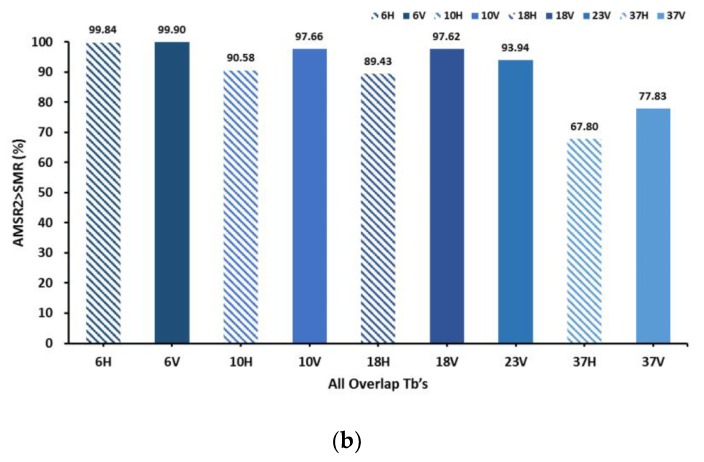
Pairwise comparison results for cold overpass times. (**a**) Average bias ofAMSR2-SMR and (**b**) percentage of AMSR2 brightness temperatures that exceed corresponding SMR observations.

A systematic bias was evident when cold overpass SMR and AMSR2 daily brightness temperatures were compared for the typical large-scope region (Figure 1) in continental Asia and continental Europe. From the distribution of scatter plots (Figure 2), the observation data of SMR and AMSR2 had great correlation, the data were basically consistent, and their distribution trends (shown by the red line in the Figure 2) were extremely close to the 1:1 line (shown by the black line in Figure 2) to different extents, respectively. The specific average deviation of observations from SMR and AMSR2 varied with frequency and polarization (Figure 3), which could demonstrate the entire average deviations and the relative differences in magnitude. A pairwise comparison of all the spatially coincident data from complementary satellite overpasses indicated that the SMR brightness temperatures were consistently colder than the corresponding AMSR2 measurements, for which Figure 3a showed specific values (AMSR2-SMR) and Figure 3b lists the complementary percentage.

The 6-V SMR brightness temperatures had an average cold deviation of 17.49 K relative to the corresponding 6-V AMSR2 brightness temperatures, which was the largest data deviation in all compared channels; similarly, for 6-H data, the cold bias was about 15.34 K, which was also obvious. Over 99.8% of the SMR 6-H brightness temperatures were less than the corresponding AMSR26-V measurement, a value that was 99.9% for the 6-V channels. Overall, the data of the 6.925 GHz frequency channels were relatively larger compared to those of the other frequencies. As for the 37-GHz observations, the average cold bias of the 37-V SMR brightness temperature was 4.04 K compared to the corresponding 37-V AMSR2 brightness temperature; at the same time, the deviation of 37-H was 2.71 K, which was the closest channel data compared to AMSR2. Correspondingly, more than 77.8% of the SMR 37-V brightness temperature was lower than the corresponding AMSR2 37-V measurement, and the result of 37-H channel was 67.8%. The deviations of horizontal and vertical polarization at 10-GHz were 5.05 K and 6.32 K, respectively; the deviations of 18-GHz were 8.55 K and 10.44 K, respectively, and the corresponding bias at 23-GHz is 8.62 K.

The results of deviation magnitude fluctuated significantly with the band frequency and slightly changed for different polarization types. At the same time, combined with the scatter plots (Figure 2) and the comprehensive comparative analysis of Figure 3b, the SMR brightness temperature results indicated a significant lower phenomenon in the study area.

### 3.2. Land Calibration for SMR

The intercalibration of satellite sensors is a typical effective way for land calibration to reduce the systematic differences between satellite sensors and to estimate the scale of any remaining uncertainty at least. The observation data that are stable in operation and well correlated to each other from SMR and AMSR2 constitute the basis for cross-calibration. For the selected pairs of brightness temperature of each channel, the linear regression was performed for the mid-latitude and high-latitude terrestrial study area (outlined in Figure 1) as the following form:

*AMSR2* (*Tb*) = *a* × *SMR* (*Tb*) + *b*, (1)

where *a* is the slope of the regression line; *b* is the intercept; and *AMSR2 (Tb)* and *SMR (Tb)* are the values of brightness temperature. The results of all slopes and intercepts calculated by linear optimal regression are summarized in Table 2.

As expected, since the configuration parameters of the two sensors are close, the slopes of the equations are close to 1, which means that the correlation between the corresponding data is high, and the intercepts in magnitude are low for each channel. Based on the regression models listed in Table 2, the brightness temperatures from SMR were calibrated for all channels. In order to facilitate the distinction, the land-calibrated brightness temperatures were marked as SMR_C. The R square between the data records from SMR and AMSR2 for each frequency and polarization was calculated separately (Table 2). It can be seen from the former scatter plots (Figure 2) that the degrees of dispersion between the vertical polarized data were smaller, which qualitatively showed that the data of vertical polarization matched better than those of the horizontal polarization. The results of R square quantitatively indicated that the correlations between the brightness temperatures of the vertically polarization were better, and the degrees of mutual matching between the corresponding data were relatively higher in the histogram.

**Figure 4:**
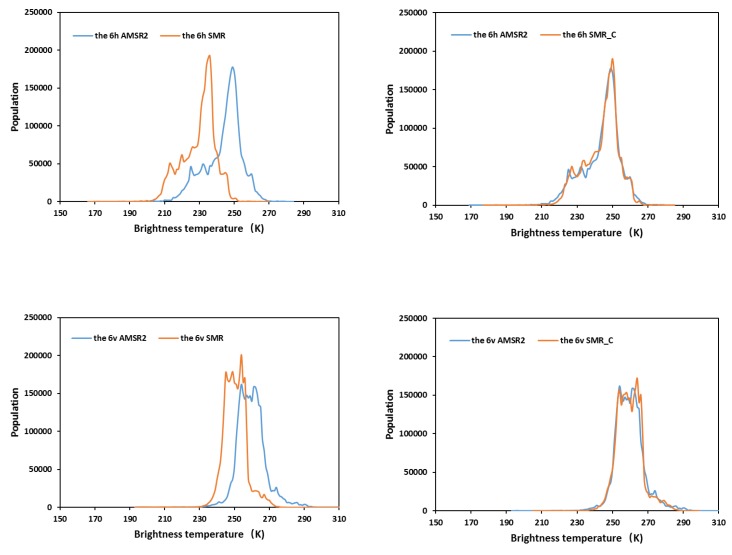

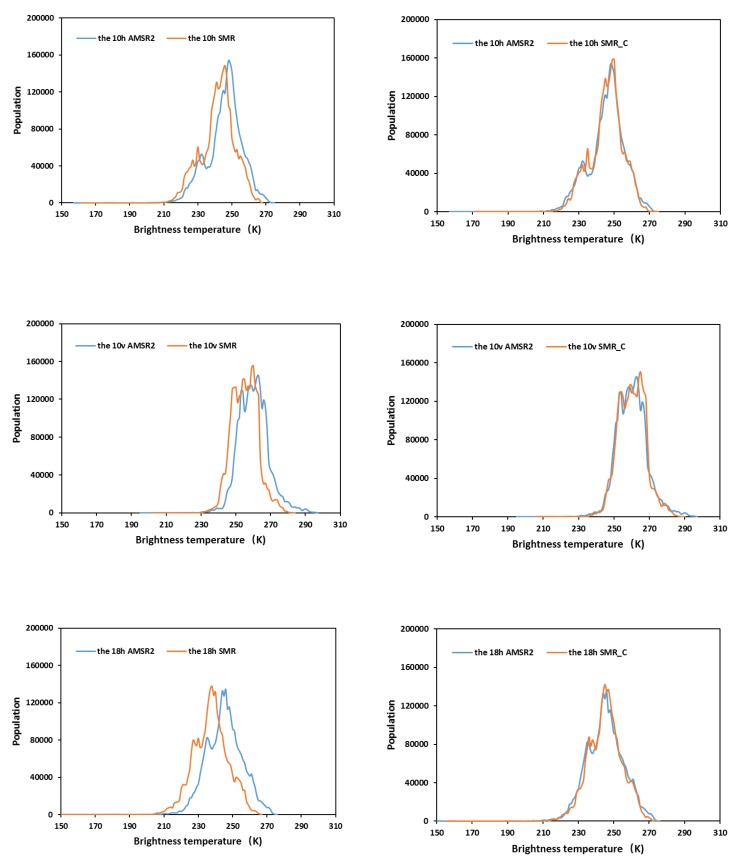

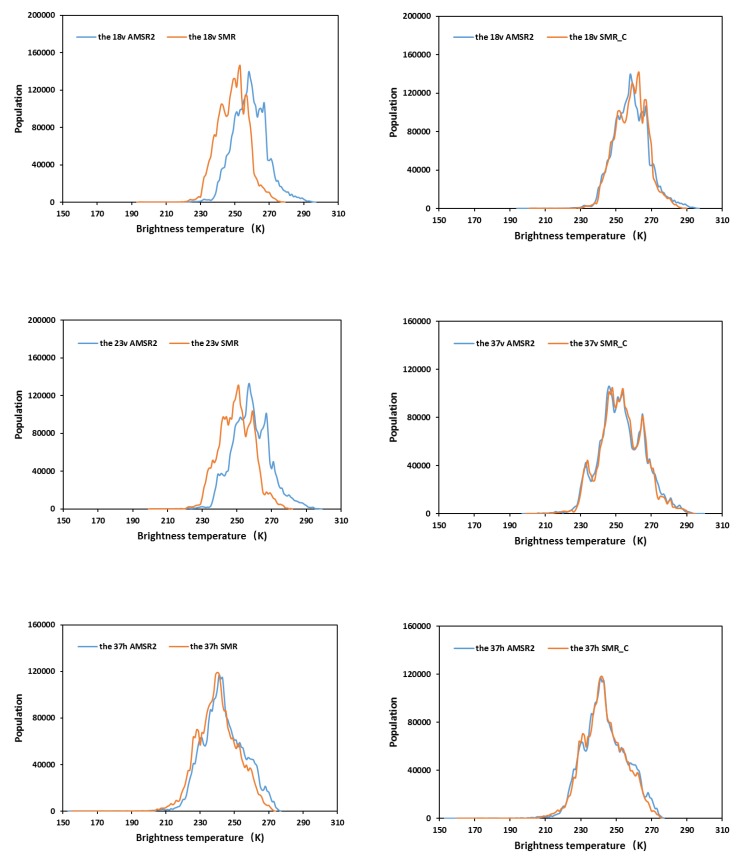

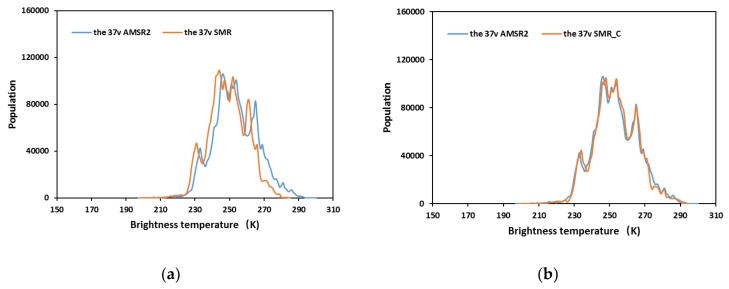
Original and Land-calibrated SMR on HY-2B and AMSR2 onboard GCOM-W1 brightness temperatures histograms during the overlapped period (from 30 October 2018 to 31 December 2018) for each channel: (**a**) SMR and AMSR2; (**b**) SMR_C and AMSR2.

The brightness temperature histograms of the SMR, AMSR2, and SMR_C for each channel (6H, 6V, 10H, 10V, 18H, 18V, 23V, 37H, and 37V, standing for 6-GHz horizontal grid data, 6-GHz vertical grid data, etc.) were generated for the entire overlapping period (Figure 4). The entire distribution showed that there was a large offset between the SMR and AMSR2 data; interestingly, all the channels of AMSR2 displayed a greater brightness temperature deviation than those of SMR. Then, the SMR brightness temperatures were adjusted by using the intercalibration coefficients in Table 2. The right subgraphs are the histograms of the adjusted SMR_C and AMSR2 data in Figure 4, which demonstrates that the intercalibration eliminates the offset. It can be concluded from the histograms that the consistency of the brightness temperature from SMR and AMSR2 can be effectively improved by cross-sensor calibration. We used the brightness temperature average biases, standard deviation (STD), and root mean square error (RMSE) as the evaluation metrics, which described the error between observations before and after land calibration. As shown in Table 3, the results indicated that all the errors of the three types after intercalibration were evidently less than those of the initial data, meaning that the land calibration can efficiently eliminate the systematic offset and improve the consistency of the observations’ data records.

### 3.3. Degree of Adjustment of the Calibration Equation

**Figure 5:**
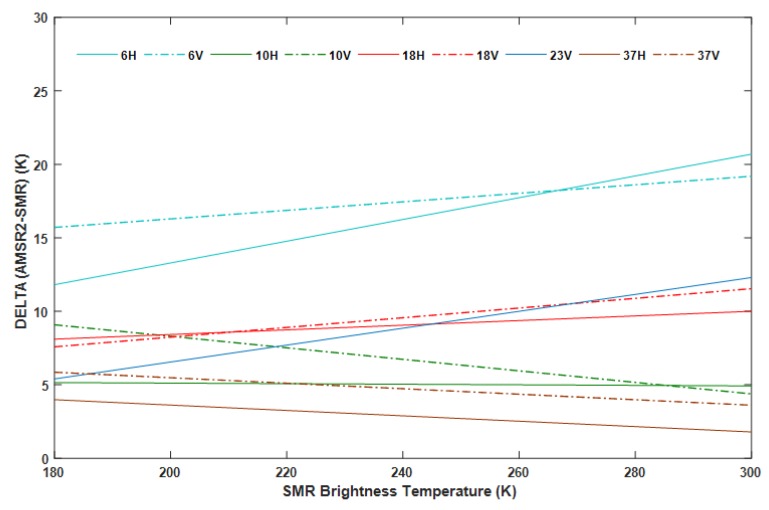
Differences between SMR and land-calibrated SMR brightness temperature data predicted by using the regression coefficients listed in Table 2 across all frequencies and polarizations.

The research results indicate that there is a large offset between the SMR and AMSR2 brightness temperature data in each channel for land surface. Meanwhile, the degree of offset varies with the frequency, polarization, and magnitude of the brightness temperature. Therefore, the land calibration must be adopted to remove the systematic deviation between SMR and AMSR2 when considering the migration of the parameter retrieval algorithms derived from AMSR2 data or combining observations from different constellations. Some researches ignored or directly used the kind of average deviation to correct the deviation and apply the data to land surface retrievals when using observations or retrieval algorithms across different data sources. Verification has shown that the snow water equivalent estimates derived from SMMR are significantly lower than those calculated using SSM/I when the system offset between datasets is not considered [5]. However, we also do not recommend average bias for modification because the relative difference between brightness temperatures from different sensors changes with the magnitude of the temperature. The degree of dispersion of points in the density scatter plot changes with the brightness temperature, which can qualitatively explain it; furthermore, the point is quantitatively demonstrated in Figure 5 where the predicted value of the deviation changes with the SMR brightness temperature. The functions that are plotted in Figure 5 are derived from the regression models in Table 2 and the expression:

*δ* = *SMR_C* (*Tb*) − *SMR* (*Tb*) = *b* − (1 − *a*) × *SMR* (*Tb*), (2)

where *SMR_C (Tb)* consists of the values of calibrated SMR brightness temperature, and *δ* is the corrected value after intercalibration. 

In Figure 5, the temperature range is from 180 to 300 K, resulting in a corresponding difference of 1.8 to 20.7 K (depending on the channel), which represents the corresponding distance from the regression line (the red line) to the 1:1 line (the black line) at different brightness temperatures in Figure 2. The difference value of δ fluctuates in the range −4.70 to 8.88 K, depending on the channel. The specific fluctuating change at the 6H, 6V, 10H, 10V, 18H, 18V, 23V, 37H, and 37V channels is 8.88 K, 3.48 K, −0.23 K, −4.70 K, 1.90 K, 3.96 K, 6.90 K, −2.20 K, and −2.36 K, respectively. Therefore, using the regression models is a significant and effective solution rather than an average deviation. On the other hand, the value of δ is the degree of correction for cross-calibration, which is more obvious and effective. Therefore, the land surface retrievals and geophysical analysis would tend to remove the impact of the relative calibration differences by using the calibrated temperatures at the channels.

## 4. Discussion

In this section, the snow extent and snow water equivalent were produced to verify the land surface application capabilities of the new SMR sensor. In order to demonstrate the effectiveness of land calibration, the consistency of the snow products derived from SMR and AMSR2 brightness temperature were then evaluated. The snow extent area and snow mass have a critical effect on biogeochemical cycling and climate change [7,39]. Passive microwave remote sensing is such an efficient method of monitoring snow that the snow extent and snow mass were produced by the brightness temperatures from before and after land calibration.

Due to the nature of the radiometer observations, the snow water equivalent retrieval is reliably shown on areas with seasonal dry snow cover. The principle of snow extent area and snow water equivalent detection is based on the volume scattering inside the snowpack [1,27]. The snow extent, based on methodology by Grody [40], utilizes a decision-tree based approach, which was recognized as the correct snow grid in our study. Due to snow and precipitation, deserts and frozen bare land have similar scattering characteristics; in order to observe snow cover area objectively, different filters were provided to separate the scattering signatures of snow cover from the other scattering signatures. The filters, produced by the brightness temperature difference of multiple frequencies, were used as the criteria for discrimination. Owing to the absence of 85 GHz with vertical polarization in SMR, one step of the criteria for frozen ground was not used. The decision tree algorithm can be described by the following relationships: for scattering signature,

*Tb*(19*V*) − *Tb*(37*V*) > 0, (3)

for precipitation,

*Tb*(22*V*) ≥ 258 *orTb*(22*V*) ≥ 165 + 0.49 × *Tb*(85*V*) *or* (258 ≥ *Tb*(22*V*) ≥ 254 *andTb*(19*V*) − *Tb*(37*V*) ≤ 2, (4)

for cold desert,

*Tb*(19*V*) − *Tb*(19*H*) ≥ 18 *andTb*(19*V*) − *Tb*(37*V*) ≤ 10, (5)

for frozen ground,

*Tb*(19*V*) − *Tb*(19*H*) ≥ 8 *andTb*(19*V*) − *Tb*(37*V*) ≤ 2 *andTb*(37*V*) − *Tb*(85*V*) ≤ 6, (6)

To avoid the effects of snow melting liquid water in the snowpack on the snow cover identification, a time-series melt-detection algorithm [41] was conducted to remove the pixels flagged as melted snow covers. The retrieval of the snow water equivalent was performed for grid cells for which dry snow cover was detected and for which snow melt was not indicated. Then, we determined the maximum and minimum of the channel difference Tb(37V) − Tb(19V), and when a certain level above the minimum was achieved, the snowmelt was considered to occur. The melting detection algorithms are as follows:

*D*(*t*) = *Tb*_37*V*_(*t*) − *Tb*_19*V*_(*t*), (7)

*D*_*max*, *tavg*_ = *max* < *D*(*t*_0_), *D*(*t*_1_), …, *D*(*t*_*N*_)>, (8)

*D*_*min*, *tavg*_ = *min* < *D*(*t*_0_), *D*(*t*_1_), …, *D*(*t*_*N*_)>, (9)

*D*(*t*) ≥ *p* × [*D*_*max*, *tavg*_ − *D*_*min*, *tavg*_] + *D*_*min*, *tavg*,_ (10)

where *D* is the channel difference, *t* is the time (in days), *p* is the level of detection (*p* = 0.9), and *N* is the averaging period *(N = 7).*

The microwave signature naturally emitted from the underlying land is weakened by the snow particles, and the weakening of the signal is related to the number of the scatterers on the radiation path, and thus to the snow depth. On the basis of this principle, the snow depth retrieval algorithm was developed by using a brightness–temperature gradient between 19 and 37 GHz [42]. Snow depth retrieval algorithms have been developed globally and regionally over the past 30 years [43,44,45]. In this study, the modified Chang algorithm [37] for the mid-latitude region in Asia was used to perform the snow depth retrieval. Then, the IGBP system classification data was used to obtain forest coverage in the grid to reduce the impact of dense forest cover.

*SD* = *a* × [*Tb*(19*H*) − *Tb*(37*H*)]/(1 − *f*), (11)

where *SD* is snow depth, *a* is the coefficient (a = 1.5), and *f* is the forest area fraction.

The constant snow density of 0.24 g/cm^3^ [46] was used in the study region. SWE can be converted from snow pressure by the following relationships:

*snow pressure* (g/cm^2^) = *snow density* (g/cm^3^) × *SD* (cm), (12)

*SWE* (mm) = *snow pressure* (g/cm^2^) × 10/*water density* (g/cm^3^), (13)

where *SD* is the snow depth and the *SWE* is the snow water equivalent. Finally, the daily snow water equivalent estimates were averaged by a seven-day sliding window to reduce the noise over the daily time series [46].

**Figure 6:**
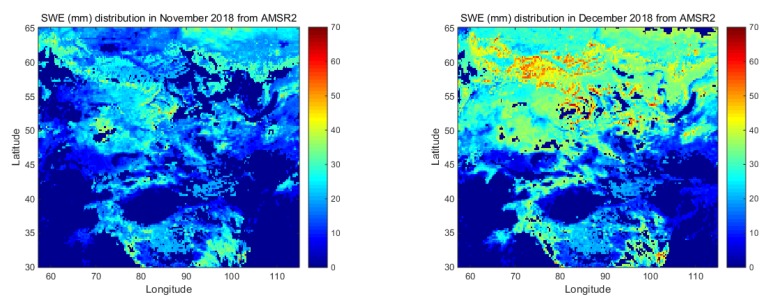

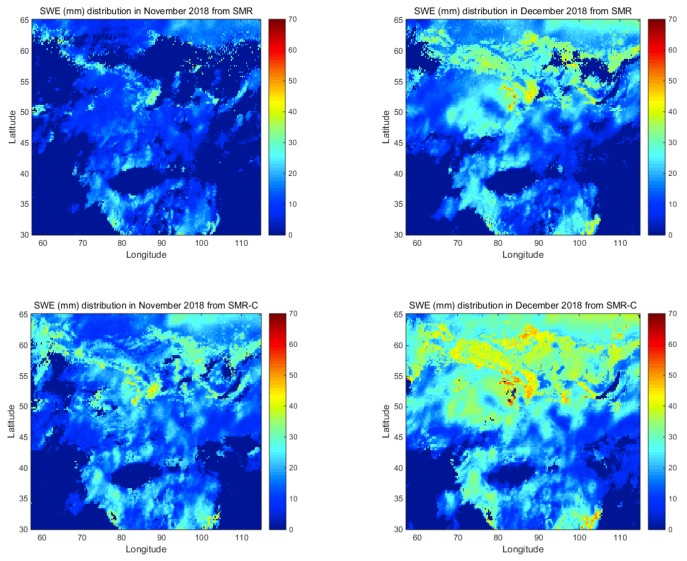
Comparison of average monthly snow depth distribution derived from the AMSR2 and SMR before and after the land-calibration operation in the overlapping period.

In this paper, we calculated the monthly snow cover area and corresponding snow water equivalent derived from the AMSR2 and SMR before and after land calibration. From the snow cover distribution shown in Figure 6, we found that distinct differences are obvious in the middle and high latitudes. Figure 6 shows the average monthly SWE distribution in November 2018 and December 2018. Based on the results from the AMSR2 (upper two), the snow extent and SWE from the initial SMR were much lower (middle two). After land calibration, the snow-cover distribution derived from SMR showed the similar spatial distribution characteristics (lower two). Therefore, we argued that terrestrial calibration effectively improves the consistency of snow water equivalent from the perspective of snow space distribution.

**Figure 7:**
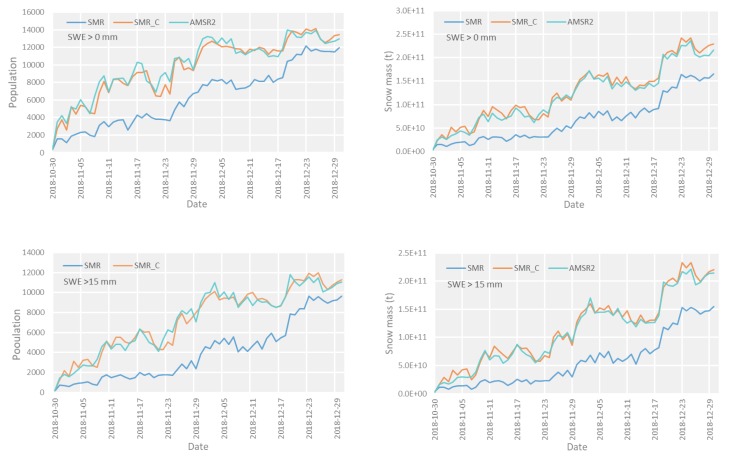

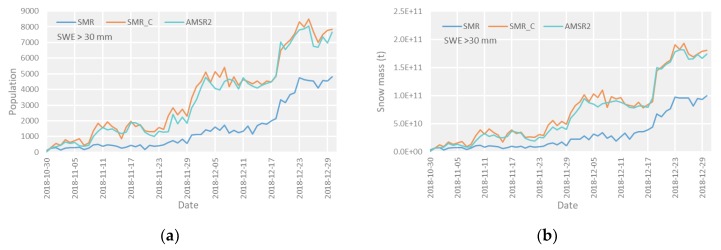
Results of the populations of the snow extent pixels and the complementary snow mass derived from SMR, SMR_C, and AMSR2 in the overlapping period at different snow water equivalent ranges (snow water equivalent >0 mm, snow water equivalent >15 mm, snow water equivalent >30 mm): (**a**) snow extent; (**b**) snow mass.

The population of the snow-extent and snow-water-equivalent grids was examined in this study using passive microwave satellite brightness temperatures from the SMR, SMR_C, and AMSR2 during the overlapping period. The snow mass was calculated based on the snow water equivalent. The population of snow-extent pixels and the total snow mass during the overlapping time are shown in Figure 7. 

In this paper, the population of the snow-cover grids was used to stand for the snow extent. The results indicated that the snow extent and snow mass derived from the initial SMR observations were much lower than those after the land calibration; however, the consistency of the results between the land-calibrated SMR data and AMSR2 was high. For a quantitative assessment of consistent results of the land calibration, the relative bias was calculated between the SMR and AMSR2, SMR_C, and AMSR2 based on the sensor of AMSR2. The relative bias was expressed as (S2−S1)/S1, where S1 represents the results of snow extent and snow mass derived from the AMSR2, and S2 represents the corresponding results of the SMR and SMR_C, respectively. As shown in Table 4, the results indicated that the extent and corresponding snow mass improved from −49.97% to 2.97% and from −51.71% to 3.01%, respectively. The absolute values of relative biases in snow extent and snow mass all decreased in the overlapping period with different snow water equivalent ranges: snow water equivalent >0 mm, snow water equivalent >15 mm, snow water equivalent >30 mm. For the snow cover area, the results of the original SMR data inversion have obvious underestimation in different snow water equivalent ranges, and the maximum deviation value can reach −59.46%. The retrieval results of the snow mass have a more obvious underestimation phenomenon, and the maximum deviation value can reach −59.97%. The relative deviation of all the inversion results under different snow water equivalent ranges is within 3.01% after land calibration. Therefore, the consistency of the snow products was improved substantially by the intercalibration for land calibration between SMR and AMSR2.

## 5. Conclusions

The SMR radiometer relies on the stable calibration of the HY-2B satellite to meet its many scientific and social application goals. The objectives of this study were to explore the radiation characteristics of the SMR and perform terrestrial calibration to serve the Earth’s surface science and climate research in mid-latitude and high-latitude regions. Focus was on the cold overpass observations, since these data are used as input to the retrieval models of geophysical parameters (discussed with snow extent area and snow water equivalent retrievals). 

In this study, the pairwise comparison of all the overlapping brightness temperature records was conducted for the spatially consistent grids. SMR Tb data were systematically lower than those of AMSR2, and the magnitude of offsets depends on channel regardless of the offsets in overpass time. We intercalibrated the brightness temperature records between SMR and AMSR2; meanwhile, we explore the potential of the SMR for monitoring land-surface parameters, and the consistency of snow extent and snow depth retrievals derived from SMR and land-calibrated SMR data was evaluated. The consistency in snow extent improved from −49.97% to 2.97%, while the consistency in snow mass improved from −51.71% to 3.01%. Cross-calibration greatly improved the consistency of the brightness temperatures from SMR and AMSR2.

In conclusion, the high correlation coefficients have demonstrated that the SMR onboard HY-2B satellite has good radiation characteristics compared to the AMSR2 and exhibits good potential for geophysical parameters retrieval. However, interestingly, all the channels of SMR present a lower brightness temperature bias than those of AMSR2. The consistency of the brightness temperatures between SMR and AMSR2 is substantially improved by land calibration, which is also for the retrievals of snow cover area and snow mass. The results of this paper are being submitted to the national data services and management of HY-2B. In the following study, we will focus on the permanent land surface calibrated models, snow product algorithms, and absolute validation of the results, expecting to offer a set of land surface quantitative productions using Chinese HY-2B satellite data.

## Figures and Tables

**Table 1:** Instrument specifications of the Scanning Microwave Radiometer (SMR) on HaiYang-2B (HY-2B) and Advanced Microwave Scanning Radiometer 2 (AMSR2) onboard Global Change Observation Mission–Water 1 (GCOM-W1).

Sensor	SMR	AMSR2
Satellite	HY-2B	GCOM-W1
Time series	October 2018 to present	July 2012 to present
Orbital altitude (km)	971	699.6
Frequency: footprint	6.925: 150 × 90	6.925/7.3: 62 × 35
Hz): (km × km)	10.7: 110 × 70	10.65: 42 × 24
	18.7: 60 × 36	18.7: 22 × 14
	23.8: 52 × 30	23.8: 19 × 11
	37: 35 × 20	36.5: 12 × 7
	/	89: 5 × 3
Polarization	V and H, except 23.8 GHz (V only)	V and H, all channels
Incidence angle (degrees)	53	55
Data acquisition	Daily	Daily
Swath width (km)	1600	1450
Inclination angle (degrees)	99.34	98.19
Orbit period (minutes)	104.5	98.8

**Table 2:** Regression slopes, intercepts values, and the R square determined by a regression between SMR and AMSR2 brightness temperature data collected over the study area.

Channel	Optimal Slope	Intercept (K)	R^2^
6H	1.0740	−1.5080	0.9012
6V	1.0290	10.4900	0.9397
10H	0.9981	5.4940	0.9195
10V	0.9608	16.1400	0.9272
18H	1.0158	5.2620	0.8354
18V	1.0330	1.6420	0.8703
23V	1.0575	−4.9500	0.8866
37H	0.9817	7.2800	0.9193
37V	0.9803	9.2210	0.9239

**Table 3:** Summary of sensor descending orbit brightness temperature average biases (unit: Kelvin), standard deviation (unit: Kelvin) values and root mean square error differences across all frequencies and polarizations data records before and after intercalibration.

Channel	Before Land Calibration			After Land Calibration		
	Bias	STD	RMSE	Bias	STD	RMSE
6H	−15.3361	3.8882	15.8213	−0.0898	2.6211	2.6436
6V	−17.4929	2.6506	17.6925	−0.0622	1.8538	1.8569
10H	−5.0521	4.0633	6.4834	−0.0097	2.7963	2.8103
10V	−6.3197	2.7753	6.9022	−0.0051	1.7187	1.7238
18H	−8.5440	7.0972	11.1086	0.0119	4.1554	4.1555
18V	−10.4377	4.8763	11.5206	0.0052	2.6857	2.6857
23V	−8.6159	5.3446	10.1389	0.0003	3.6688	3.6404
37H	−2.7124	7.2239	7.7163	0.0105	4.1768	4.1768
37V	−4.0435	6.0908	7.3108	0.0050	3.3378	3.3378

**Table 4:** Relative bias for the population and snow mass derived from the SMR data before and after land calibration for different snow water equivalent extends.

	Before Land Calibration		After Land Calibration	
	Population	Snow Mass	Population	Snow Mass
SWE > 0 mm	−49.97%	−51.71%	2.97%	3.01%
SWE > 15 mm	−53.65%	−55.92%	1.99%	1.27%
SWE > 30 mm	−59.46%	−59.97%	2.98%	2.51%
